# Supplementary material for: Heparanase Expression Propagates Liver Damage in CCL4-Induced Mouse Model
Source: Cells. 2022 Jun 27;11(13):2035. doi: 10.3390/cells11132035 (PMC9265342; doi:10.3390/cells11132035)
Supplement: Supplementary file 1 [file cells-11-02035-s001.zip › cells-1763296-supplementary.pdf]

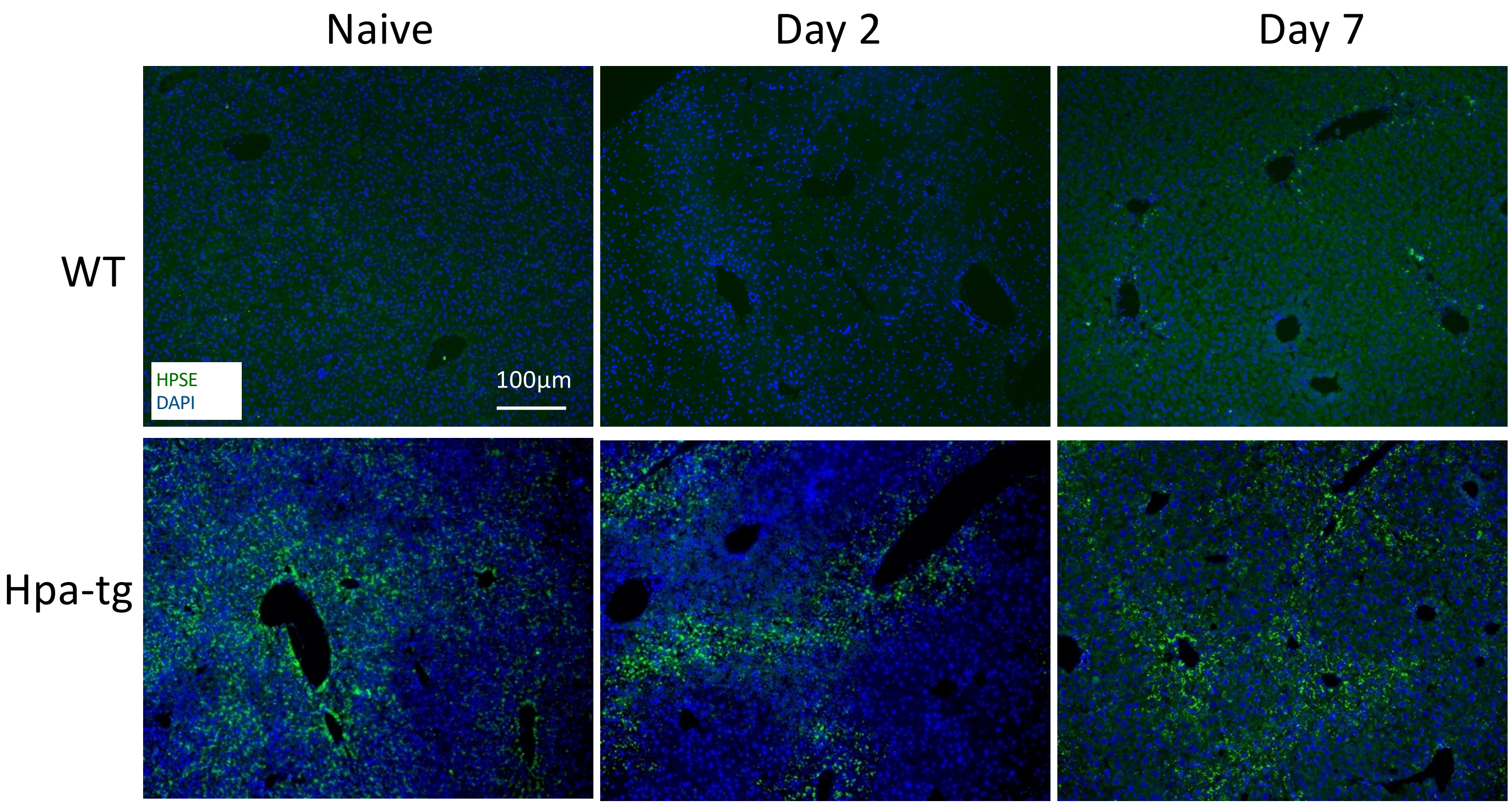

Figure S1. Immunohistostaining of paraffin-embedded sections showing overexpression of heparanase in Hpa-tg liver tissues. Heparanase expression is slightly elevated in WT liver 7 days after CCL4 induction.

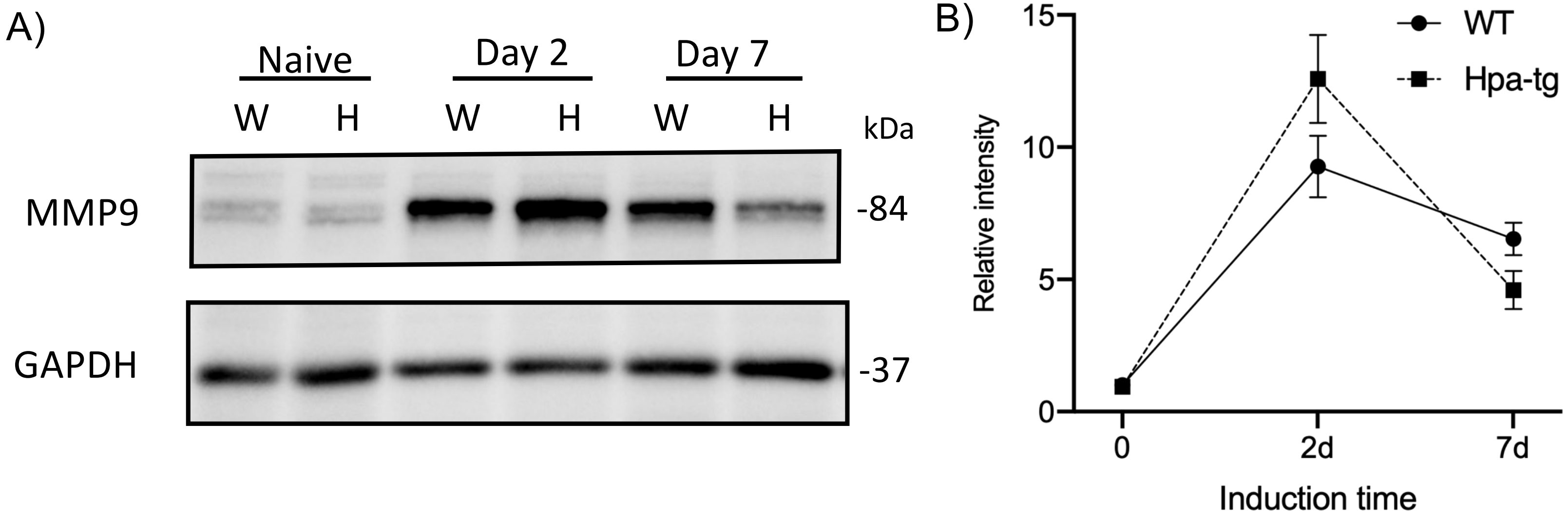

Figure S2. Western blot analysis of MMP9 in liver tissue lysates derived from naïve and CCL4-treated (2 and 7 days) WT (W) and Hpa-tg (H) mice. Frozen liver tissues were homogenized in RIPA buffer and protein was quantified. Mixed lysates (40µg protein) from 5-6 mouse livers were applied onto 8% SDS-PAGE and probed with anti-MMP9 antibody. (**A**) Representative Western blot; (**B**) Band intensity was measured by IMAG LAB software and normalized by the intensity of GAPDH. Naïve WT value was set arbitrarily to a value of 1. Data are expressed as mean$\pm$SEM of bands scanned from 6 WB blots.

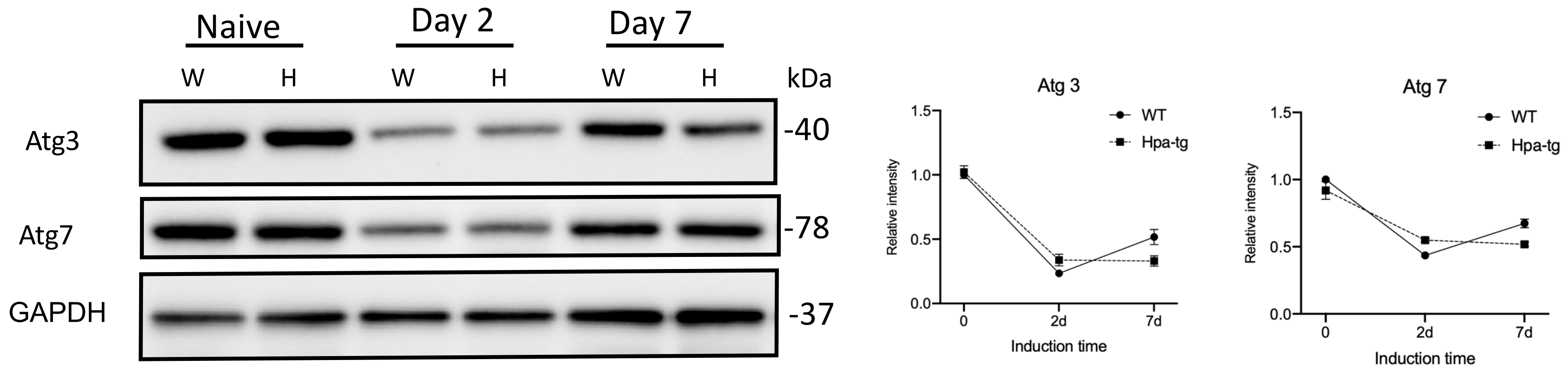

Figure S3. Western blot analysis of liver tissue lysates derived from naïve and CCL4-treated (2 and 7 days) WT (W) and Hpa-tg (H) mice. Frozen liver tissues were homogenized in RIPA buffer and protein was quantified. Mixed lysates (40 µg protein) of 5-6 mouse livers were applied onto 10% SDS-PAGE and probed with antibodies directed against Atg3, Atg4 and GAPDH. (**A**) Representative Western blot; (**B**) Band intensity was quantified by IMAG LAB software and normalized by the intensity of GAPDH. Naïve WT value was set arbitrarily to a value of 1. Data are expressed as mean±SEM of bands scanned from 4 WB blots.

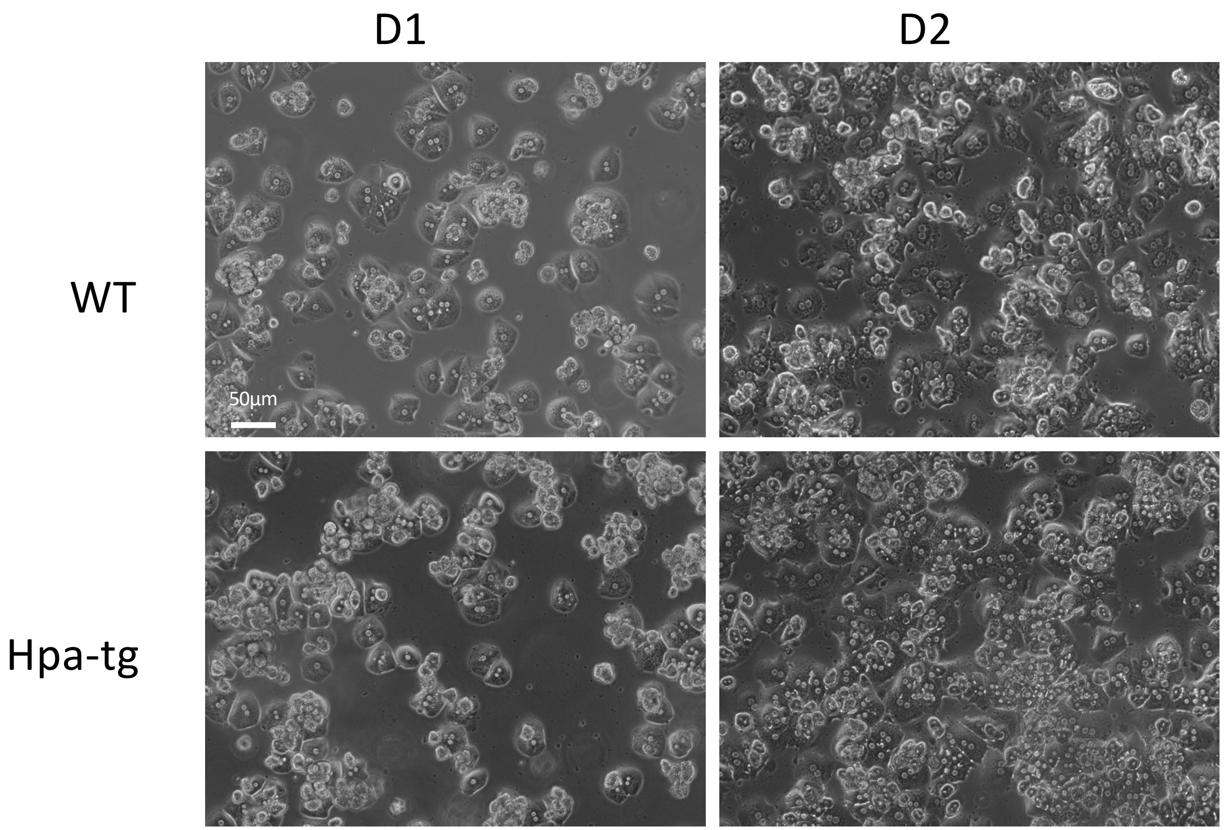

Figure S4. Hepatocytes were isolated from naïve mouse liver and $0.5x10^6$ cells were seeded into 6-cm plates. Images taken on days 1 and 2 show no morphological differences between WT and Hpa-tg mice.

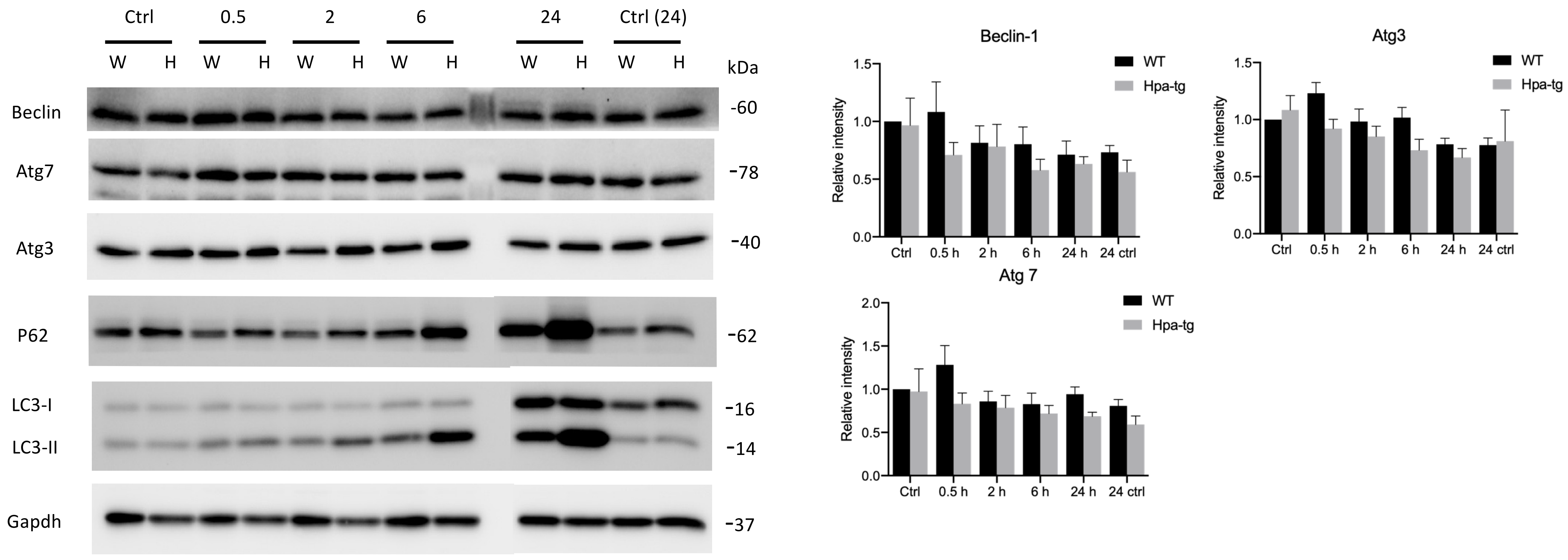

Figure S5. Western blot analysis of hepatocyte cell lysates. Cells derived from WT (W) amd Hpa-tg (H) livers were cultured in serum-free medium for 16.5 h (starvation). Chloroquine (CQ) was then added to a final concentration of 20 μM. Cells were collected at the indicated time points (h) and lysed in RIPA buffer. The supernatants (40 μg total protein) were analyzed by Western blotting. (**A**) Representative blot. (**B**) Band intensity was measured by Image LAB software and normalized to GAPDH. The relative band intensity of naïve WT cells prior to addition of CQ (Ctrl) is defined as 1. Each bar represents the average intensity of cell lysates originating from 5 mice. Ctrl (24) represents cells cultured under the same conditions for 24 h without CQ.

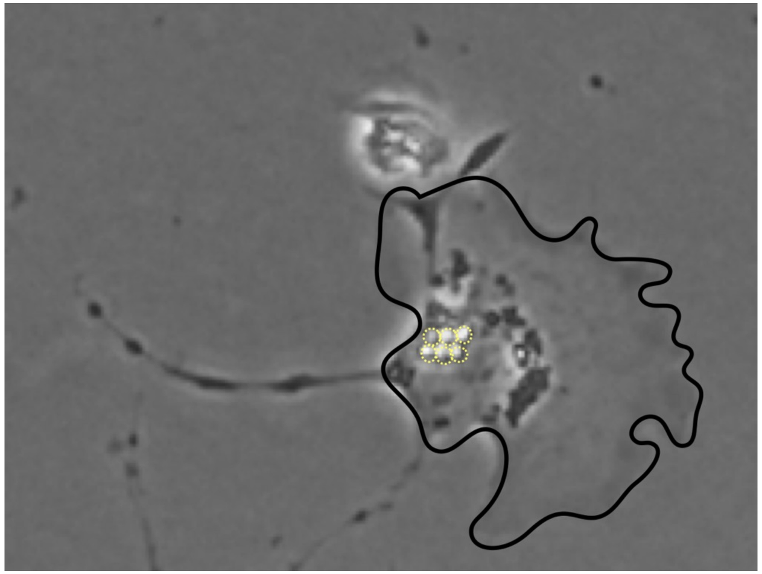

Figure S6. The cell area was defined by outlining (Image J) the morphological edges of each cell.
